# Study of Mitogenomes Provides Implications for the Phylogenetics and Evolution of the Infraorder Muscomorpha in Diptera

**DOI:** 10.1002/ece3.70832

**Published:** 2025-01-16

**Authors:** Huan Yuan, Wenbo Fu, Shulin He, Tingjing Li, Bin Chen

**Affiliations:** ^1^ Chongqing Key Laboratory of Vector Control and Utinization; Institute of Entomology and Molecular Biology, College of Life Sciences Chongqing Normal University Chongqing China

**Keywords:** Diptera, evolution, mitogenome, Muscomorpha, phylogenetics

## Abstract

The Muscomorpha is one of the most species‐rich brachyceran groups in Diptera, with many species serving as important disease vectors; however, its high‐level phylogenetic relationships have long been controversial and unsolved. This study comparatively analyzed the characteristics of mitogenomes of 131 species that represent 18 superfamilies in Muscomorpha, in which mitogenomes of 16 species have been newly sequenced and annotated, demonstrating that their gene composition, order, AT bias, length variation, and codon usage are consistent with documented dipteran mitogenomes. The phylogenetic topologies demonstrated that the robustness of Muscomorpha and major clades within Muscomorpha are monophyletic: Cyclorrhapha, Schizophora, and Calyptratae. A clade of Empidoidea were recovered as the sister group to Cyclorrhapha. Within Cyclorrhapha, Platypezoidea and Syrphoidea were sequentially placed as basal groups of the Cyclorrhapha. The remaining cyclorrhaph superfamilies gathered as two main clades. Ephydroidea were, in most cases, placed as the sister group to Calyptratae. Within Calyptratae, Hippoboscoidea were sister to an assemblage of lineages composed of an Oestroid grade and Muscoidea. The Muscomorpha was proposed to originate in the early Jurassic, and the main clade diversified near the Cretaceous–Paleogene extinction event, estimated using the MCMCtree and six fossil calibration points. The ancestral area of origin and geographic range of Muscomorpha was deduced to be the Palaearctic region with 56.9% probability using the RASP software based on a dated tree.

## Graphical abstract

This study comparatively analyzed the mitogenome characteristics of 131 species that represent 18 superfamilies in Muscomorpha, in which mitogenomes of 16 species are newly sequenced and annotated herein. Further, we deduced their phylogenetics using maximum likelihood, and discussed their divergence time and evolution combined with taxonomy and historical geography. This is the first comprehensive work on characteristics of mitogenome, and mitogenome‐based phylogenetics and evolution in Muscomorpha, which lays an important base for further study on mitogenomics and systematics of Muscomorpha.

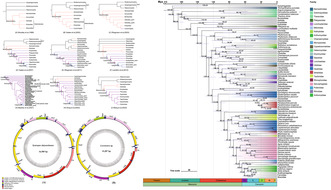

## 1 Introduction

Muscomoropha, known as the higher fly within Diptera, constitutes an infraorder in Brachycera. There are over 100,000 species documented worldwide with diverse ecological roles (Wiegmann, Mitter, and Thompson 1993). This infraorder includes several common groups, such as horse flies, bat flies, snipe flies, robber flies, house flies, flesh flies, blow flies, and bottle flies. Most of them can transmit a variety of pathogens to humans and many vertebrates due to their hematophagy (e.g., Calliphoridae, Oestridae), parasitoids (e.g., Tachinidae, Gasterophilinae) and predation (e.g., Asilidae) habits. Housefly is involved in the transmission of over 30 bacteria, potozoa, viruses, and helminth eggs (Barin et al. 2010; Al‐Enazi et al. 2018); some flies cause incalculable economic damage to plants due to their phytophagous (e.g., Tephritidae, Agromyzidae) and xylophagy (e.g., Xylophagidae, Syrphidae). Quarantine and eradication programs cost tens of millions of dollars annually in some areas in response to the threat of flies to agriculture (Daane and Johnson 2010). Although Muscomoropha has high economic, sanitary, medical, and ecological importance, its high‐level phylogenetic relationships and evolution have long been controversial and unsolved, which has seriously hindered the construction of taxonomic system.

**FIGURE 1:**
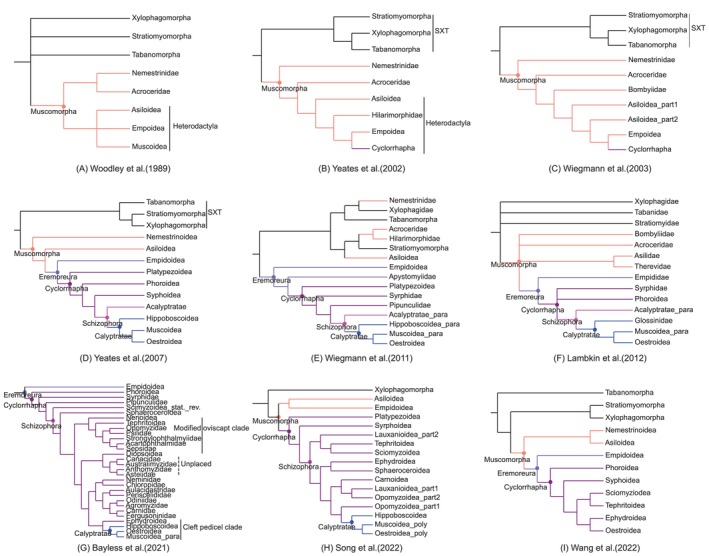
Phylogenetic hypotheses were suggested in earlier studies including Muscomorpha. (A) Woodley (1989) based on morphological data; (B) Yeates (2002) based on morphological data; (C) Wiegmann et al. (2003) based on morphological and molecular data; (D) Yeates et al. (2007) based on morphological and molecular data; (E) Wiegmann et al. (2011) based on morphological and molecular data; (F) Lambkin et al. (2012) based on morphological data; (G) Bayless et al. (2021) based on transcriptomic data; (H) Song, Xi, and Yin (2022) based on mitogenomic data; (I) Wang et al. (2022) based on mitogenomic data.

The taxonomy of the infraorder Muscomorpha can be traced back to the middle of the 20th century (Crampton 1944). The characterization of the Muscomorpha (= Eremoneura) was discussed by Hennig (Hennig 1952), who classified the Cyclorrhapha and Orthogenya (= Empidiformia) in this group (Griffiths 1972). Woodley (1989) extended the definition of Muscomorpha to include Nemestrinoidea and Heterodactyla (= Asiloidea + Eremoneura) (Woodley 1989) (Figure 1A). Subsequently, an alternative classification including Cyclorrhapha into the infraorder was proposed, which divided the Cyclorrhapha into Aschiza and the Schizophora sections; the latter section can be divided into two subsections. The Acalyptratae and Calyptratae are commonly referred to as acalyptrate muscoids and calyptrate muscoids, respectively (Yeates and Wiegmann 1999). In this study, we adopt the nomenclature proposed by Woodley in his Manual Nearctic Diptera Volume 3, whereby the infraorder Muscomorpha is defined as comprising all brachyceran families, except those belonging to Stratiomyomorpha, Xylophagomorpha, and Tabanomorpha (Woodley 1989). Numerous phylogenetic studies have investigated the relationships involving the infraorder level, employing a variety of methodologies and datasets. However, the relationships within the infraorder remain a subject of ongoing debate. Prior to and during the early 21st century, dipterists primarily relied on morphological data to elucidate phylogenetic relationships (Yeates and Wiegmann 1999; Yeates 2002; Yeates et al. 2007). Yeates et al. (2007) synthesized these phylogenetic relationships using supertree analysis, which provided a phylogenetic framework for the infraorder: Nemestrinoidea + (Asiloidea + (Empidoidea + (Platypezoidea + (Phoroidea + (Syrphoidea + Schizophora))))) (Yeates et al. 2007) (Figure 1D). Nemestrinoidea and Asiloidea are placed sequentially as basal groups of the infraorder and as closest relatives of Eremoneura (Empidoidea + Cyclorrhapha) (Figure 1A,B,D). Nemestrinidae and Acroceridae have been united into Nemestrinoidea because of their parasitic larvae (Woodley 1989) (Figure 1A). Subsequent phylogenetic results proved that Nemestrinidae is a monotypic family in Nemestrinoidea at the basal of the infraorder (Yeates 2002; Wiegmann et al. 2003) (Figure 1B,C). In contrast to earlier research based solely on molecular data, recent studies integrating both molecular and morphological data indicate that Nemestrinidae, Acroceridae, and the monophyletic Asiloidea are as three separate clades nested within the SXT clade, rather than being positioned as basal groups of Muscomorpha (Wiegmann et al. 2011) (Figure 1E), Additionally, another molecular estimate of higher‐level Brachycera phylogeny reveals that Nemestrinidae and Acroceridae are united together as the closest relatives to the SXT clade (Shin et al. 2017). The families Asilidae, Apioceridae, Mydidae, Scenopinidae, Therevidae, and Bombyliidae have been classified under the superfamily Asiloidea based on the apomorphic trait of having the posterior spiracle located in the apparent penultimate segment of the abdomen (Woodley 1989; Yeates 2002). But later same type but mass evidence has failed to recover the monophyly of Asiloidea, nor have clarified the phylogenetic relationships among its constituent members (Lambkin et al. 2012) (Figure 1F). Even more perplexing is the fact that Asiloidea, which was shown to be monophyletic under the combined evidence, is more closely related to Stratiomyomorpha (Wiegmann et al. 2011) (Figure 1E). Although recent molecular evidence attempts to resolve the phylogenetic relationships at the suborder level (Shin et al. 2017; Song, Xi, and Yin 2022; Wang et al. 2022), there still no universal consensus on who lies at the base of Muscomorpha near the Eremoneura. A major of phylogenetic scrutinies have reached a consensus regarding the large infraordinal grade: Eremoneura (Figure 1B–G,I). The Empidoidea are consistently recognized as monophyletic and a sister to the Cyclorrhapha (higher flies) (Yeates 2002; Wiegmann et al. 2003; Yeates et al. 2007; Lambkin et al. 2012; Bayless et al. 2021; Wang et al. 2022). Notwithstanding this, due to the sampling of monotypic Apystomyiidae, the Empidoidea is no longer as the sister group to the Cyclorrhapha (Wiegmann et al. 2011; Shin et al. 2017) (Figure 1E). Another competing relationship, based on recent mitogenomic work, indicates that the Empidoidea clusters with the Asiloidea, forming a sister relationship with the Cyclorrhapha (Song, Xi, and Yin 2022) (Figure 1H). Investigating the closest relatives of the Cyclorrhapha remains a challenge in the era of the molecular phylogenetics.

The monophyletic Cyclorrhapha (Platypezoidea + (Phoroidea + (Syrphoidea + Schizophora))) is well supported by the supertree analysis (Yeates et al. 2007). The Platypezoidea (Platypezidae, Lonchopteridae, Ironomyiidae, Sciadoceridae, and Phoridae) and the Syrphoidea (Syrphidae and Pipunculidae) are monophyletic based on the fused condition of the hypopharyngeal and as sister group based on tentoropharyngeai sclerites in the larvae of both superfamilies (McAlpine and Wood 1989). Notwithstanding this characteristic, recent studies concerning the nomenclature of the Platypezoidea and Phoroidea have introduced additional confusion. Initially, based on morphological characters, Phoridae was proposed as a sister group to Lonchopteridae, forming a clade referred to as Phoroidea (Lambkin et al. 2012). Subsequent phylotranscriptomic analysis also included Platypezidae within Phoroidea too (Bayless et al. 2021). Conversely, Wiegmann categorized Platypezidae, Lonchopteridae, Phoridae, Ironomyiidae, and Sciadoceridae within a clade designated as Platypezoidea (Wiegmann et al. 2011), and a more recent mitogenomic analysis only included Phoridae and Lonchopteridae united together as Platypezoidea (Song, Xi, and Yin 2022). Consequently, these conflict conclusions have led to confusion surrounding the nomenclature of Platypezoidea and Phoroidea. In the other hand, the monophyly and relationships of the three lineages Platypezoidea, Phoroidea and Syrphoidea as lower cyclorrhaphan grade previously referred to as Aschiza (non‐schizophoran families in Cyclorrhapha) has long been lacking both robust morphological (Lambkin et al. 2012) and molecular support (Wiegmann et al. 2011; Bayless et al. 2021; Song, Xi, and Yin 2022; Wang et al. 2022) (Figure 1E–I). Such the monophyly of Syrphoidea holds in a series of analyses based on morphological data (Yeates and Wiegmann 1999; Yeates 2002; Yeates et al. 2007), but results inferred from molecular data break up the Syrphoidea with the Syrphidae and Pipunculidae (Wiegmann et al. 2011; Bayless et al. 2021; Song, Xi, and Yin 2022).

The Schizophora comprise just over half of the family‐level diversity in Diptera (approximately 80 families) that is generally accepted as monophyletic (McAlpine and Wood 1989). Traditionally, Schizophora as a clade has been divided into two groups: Acalyptratae and Calytratae; McAlpine based morphological features and Junqueira applied poor‐samplings mitogenomic data proposed that Acalyptratae and Calytratae are monophyletic separately (McAlpine 1989; Junqueira et al. 2004). Subsequent morphological and molecular investigations denied the Acalytratae are monophyletic but rather detected as a paraphyletic assemblage (Lambkin et al. 2012; Wiegmann et al. 2011; Song, Xi, and Yin 2022). The Calytratae are certainly monophyletic (Lambkin et al. 2012; Wiegmann et al. 2011; Song, Xi, and Yin 2022; Kutty et al. 2019) comprised of three distinct superfamilies: Hippoboscoidea, Oestrioidea, and Muscoidea (Kutty et al. 2010; Yan, Pei, and Zhang 2021; Nirmala, Hypša, and Žurovec 2001). Although monophyly is determined, the relationships of schizophoran lineages are contentious in studies employing morphological traits and molecular data (Lambkin et al. 2012; Wiegmann et al. 2011; Bayless et al. 2021). Because the number and relationships of the component families even superfamilies for phylogenetic workers are too variable to recognize them, such questions to which clade is first diverged branch within Schizophora, which clade is the sister of the undoubtedly monophyletic Calyptratae. The morphological work attested the relationships among 17 families (seven from Calyptratae) among Schizophora. Conopidae was the first to branch of this group, Tephritidae was supported as a sister group to Calyptratae (Lambkin et al. 2012); The comprehensive phylogenetic supported the monophyly of five superfamilies in the Acalyptratae (Tephritoidea, Nerioidea, Lauxanioidea, Sciomyzoidea, Ephydroidea) and Oestroidea in the Calyptratae, Ephydroidea is the sister to Calyptratae (Wiegmann et al. 2011); A recent transcriptome‐based phylogenomic analysis is the most research involving 46 families to address the phylogeny of Schizophora (Bayless et al. 2021), its results supported Sciomyzoidea is the first branch of Schizophora and Ephydroidea is the sister to Calyptratae; A recent mitogenome‐based phylogenomic analysis involving 22 families of Schizophora, in most cases, grouped Sphaeroceroidea and Ephydroidea as the sister group to Calyptratae, but in this work different datasets and phylogenetic inference methods lead to inconsistent results (Song, Xi, and Yin 2022). All the resolution of relationships among schizophoran groups indicated that there remains a major challenge for its phylogenetics.

The fossils of the Eremoneura are found in the Cretaceous (Grimaldi and Cumming 1999; Grimaldi and Engel 2005) and well‐preserved tabanids, nemestrinids, bombyliids and mydids have been recovered from the Upper Jurassic (Ren 1998). Divergence times inferred from 28S rDNA and some several fossils suggest that Muscomorpha likely originated around the same period as the earliest estimates for angiosperms. Its diversification is marked by the emergence of Asiloidea during the Jurassic period (approximately 200–170 Mya) and reaches its peak within the Schizophora during the Tertiary period, specifically between 65 and 20 Mya (Wiegmann et al. 2003). The molecular‐based time‐calibrated phylogeny of Dipteran families has also demonstrated that the lower Brachycera rapidly radiated in the mid‐Jurassic (~180 Mya). The origins and diversification of the major lineages of Muscomorpha are likely to be much more recent (Wiegmann et al. 2011). Despite there being so many works estimating divergence dates of the major lower brachyceran fly lineages now, the accurate age estimates among the major Muscomorpha lineages have been problematic or vague because of the absence of consistent evidence and the rarity of well‐preserved fossils.

The mitochondrion is a critical organelle in eukaryotic cells and has a small genome called the mitochondrial genome (mitogenome), as a typical mitogenome, *Drosophila yakuba* (Clary and Wolstenholme 1985). The length of Diptera mitogenomes is mostly 14–20 kb, including 37 genes—13 protein‐coding genes (13 PCGs, ≈75% of the genome), 22 transfer RNA (22 tRNAs, ≈10%) genes, two ribosomal RNA (2 rRNAs, ≈15%) genes and a variable control region (CR), and these genes are structured in a compact circular genome, which has the advantageous features of maternal inheritance and a low level of recombination (Cameron 2014). Mitogenomes have been evaluated as a compelling tool for understanding phylogenetic relationships within many insect groups, such as Coleoptera (Yuan et al. 2016), Hymenoptera (Tang et al. 2019), Hemiptera (Li et al. 2017; Du et al. 2019; Zhao et al. 2019), and Lepidoptera (Timmermans, Lees, and Simonsen 2014). As of 19 March 2023, 376 complete or nearly complete mitogenomes of Muscomorpha have been submitted to the NCBI database. The mitogenomics of Diptera has also been extensively studied (Guo et al. 2021; Zhang et al. 2019). Two of the most comprehensive mitogenome studies focus on Nematocera (Zhang, Yang, and Kang 2023) and Branchycera (Song, Xi, and Yin 2022), which included 116 and 187 dipteran representatives, respectively. Within the Branchycera, many major groups have been touched yet the available research on Muscomorpha precludes a comprehensive examination of phylogenetic relationships using mitogenome data (Pu et al. 2017). Furthermore, the monophyletic status and intrafamilial relationships of many members remain to be elucidated.

In this study, we sequenced and annotated mitogenomes of 16 species, and comparatively analyzed the mitogenome characteristics of Muscomoropha based on 131 species covering 18 superfamilies. More importantly, we deduced and discussed the phylogenetics based on mitogenomes, and estimated the divergence time of major phylogenetic nodes using incorporating six fossil records as references. This is the first comprehensive study on the characteristics of mitogenomes, and mitogenome‐based phylogenetics and evolution in the infraorder, which lays an important base for further study on mitogenomics and systematics of the infraorder.

## 2 Materials and Methods

### 2.1 Insect Sampling and Sequencing

Sixteen species of samples from 11 families were collected from Chengkou County in Chongqing (Table S1). All samples were collected from the field and did not require any permits. The collected speciesmens were preserved in 95%–100% ethanol and stored in a −20°C freezer. All were morphologically identified by dipterists and verified using *COX1* sequencing. A total amount of 0.2 μg DNA per sample with 40 ng/μL of was extracted from the thoracic muscle tissues of a single adult specimen using the Qiagen Genomic DNA Kit (Qiagen, Duesseldorf, Germany). A paired‐end library of 350 bp was constructed, and high‐throughput sequencing was performed on the Illumina Hiseq X10 sequencer, achieving a sequencing depth of 100× for the samples. Consequently, over 10 Gb of raw data were generated for each sample. High‐quality reads were obtained by removing adapters and poly‐N sequences, and reads with more than 50% low quality bases (Q ≤ 5) were filtered out using FastQC (http://www.bioinformatics.babraham.ac.uk/projects/fastqc/), clean mitogenomic reads were extracted by Basic Local Alignment Search Tool (https://blast.ncbi.nlm.nih.gov/Blast.cgi) search against the read pool with known relative sequences as query sequences. Next, a mitogenome assembly was performed with NOVOPlasty version 2.6.2 (Dierckxsens, Mardulyn, and Smits 2017).

### 2.2 Mitogenome Annotation and Characteristics Analysis

Initial annotation of these sequences was performed on the Mitos web server (Meng et al. 2019). The open reading frames further were manually corrected by Geneious v 4.8.5 (Kearse et al. 2012) with the invertebrate mitochondrial codon table, and compared with other homologous species using blastp and the nr database. The tRNA genes were submitted to the tRNAscan‐SE (http://lowelab.ucsc.edu/tRNAscan‐SE/) for correction and prediction of a secondary structure. And the complete mitogenome was finally visualized on the CGView Server (http://stothard.afns.ualberta.ca/cgview_server/) (Grant, Arantes, and Stothard 2012). AT‐skew [(A − T)/(A + T)] and GC‐skew [(G − C)/(G + C)] were estimated to investigate nucleotide composition bias (Perna and Kocher 1995), and scatterplots of AT‐Skew, GC‐Skew and AT% were drawn using python. The selection pressure of the 13 PCGs was analyzed by calculating Ka (non‐synonymous mutation rates) and Ks (synonymous mutation rates) values with DnaSP v6.11.1 (Rozas et al. 2017).

### 2.3 Matrix Generation and Phylogenetic Analysis

Our phylogenetic analysis included published sequences from 131 muscomorpha, representing 53 families from 18 superfamilies, with three Xylophagaidae species ( *Coenomyia ferruginea* , *Dialysis* sp., *Heterostomus* sp.) as outgroups (all sequences were downloaded on March 19, 2024) (Table S2). Multiple sequence alignment precedes matrix generation, we employed the codon‐aware program MACSE v2.06 for 13 PCGs and MAFFT version 7.0 with the G‐INS‐i strategy for 2 rRNAs (Katoh and Standley 2013), thereafter, the 13 PCGs were subjected to trimming using Gblocks under the invertebrate mitochondrial genetic code (Talavera and Castresana 2007), while the two rRNA sequences underwent trimming using trimAl v1.2rev57, subsequently, all individual alignments were concatenated into a supermatrix using the Phylosuite_v1.2.3 platform with default settings (Xiang et al. 2023; Zhang et al. 2020). We constructed 4 datasets for phylogenetic analyses: (1) PCGsrRNA, the combination of 13 protein‐coding genes plus two rRNA genes, resulting in a total sequence length of 12,641 nucleotides; (2) PCGs12rRNA, to mitigate substitution saturation, the third codon positions of 13 PCGs were excluded; (3) PCGs, all codon positions; and (4) AA, amino acids translated by PCGs. Before phylogenetic analyses, the substitution saturation of each codon position of the 13 mitochondrial PCGs was assessed using the index (Iss) with DAMBE v.6 (Xia 2017). The completeness of multiple sequence alignments was quantified by AliStat (Wong et al. 2020), and the heterogeneity of sequence was visualized using AliGROOVE v.1.08 (Kück et al. 2014). To determine the optimal partitioning schemes and corresponding nucleotide substitution models for each dataset, we employed ModelFinder to select the best‐fit substitution model for each partition in maximum likelihood (ML) analysis. To avoid the influence of heterotachous evolutionary sequences on phylogenetic inference, we used the single topology (GHOST) model in IQ‐TREE (Minh et al. 2020), the Bayesian information criterion (BIC) and the ‘greedy’ algorithm were used, with branch lengths estimated as ‘unlinked’, to search for the best‐fit scheme in the partition model. To mitigate the effects of long‐branch attraction (LBA) artifacts, the posterior mean site frequency (PMSF) model was manipulated in IQ‐TREE too. In the concatenated analyses, support values were assessed using the ultrafast bootstrap (UFBoot), the approximate likelihood ratio test (SH‐aLRT), and a Bayes test (Anisimova et al. 2011).

### 2.4 Divergence Time Estimates and Biogeographical Analyses

Divergence time analyses were conducted using the MCMC Tree in PAML v4.9j, with six soft fossil constraints (Table S3) under the GTR molecular clock model (Yang 2007). To reduce the computational burden, approximate likelihood calculation and ML estimation of branch lengths were employed. Hessian matrices were calculated using the GTR substitution model and the independent rates clock model. The preferred topology estimated from partition ML analysis was selected as the input tree. A total of 20,000 iterations were burn‐in, with sampling occurring every 10 iterations until 5000 samples were gathered. Further details regarding parameter settings, calibration points, and MCMC runs can be found in the control file. The convergence of the MCMC runs was assessed based on convergence and infinite‐sites plots, following the guidelines provided in the package manual. For each analysis, we employed the FigTree and TVBOT online tools (https://www.chiplot.online/tvbot.html) to visualize the phylogenetic tree and the corresponding branch lengths.

We inferred historical biogeography using the R package ‘BioGeoBEARS’ (Matzke 2014) implemented in RASP 4 (Yu, Blair, and He 2020) with time‐calibrated phylogenetic tree without outgroups as input. The distributional data of the taxon were collected from the Global Biodiversity Information Facility database (https://www.gbif.org/) and some taxonomic references (Table S4). Based on relevant previous biogeographic studies (Yan, Buenaventura, et al. 2021), six biogeographic regions were used: (A) Afrotropical region, (B) Palaearctic region, (C) Oriental region, (D) Australasian, (E) Nearctic region and (F) Neotropical region. Maximum range‐size was set to six because living species of Muscomorpha are distributed worldwide. The BAYAREALIKE+J model with the highest Akaike Information Criterion weight (AICc_wt) was chosen as the most suitable model (Table S5).

## 3 Results

### 3.1 Mitogenome Nucleotide Composition and Organization

**FIGURE 2:**
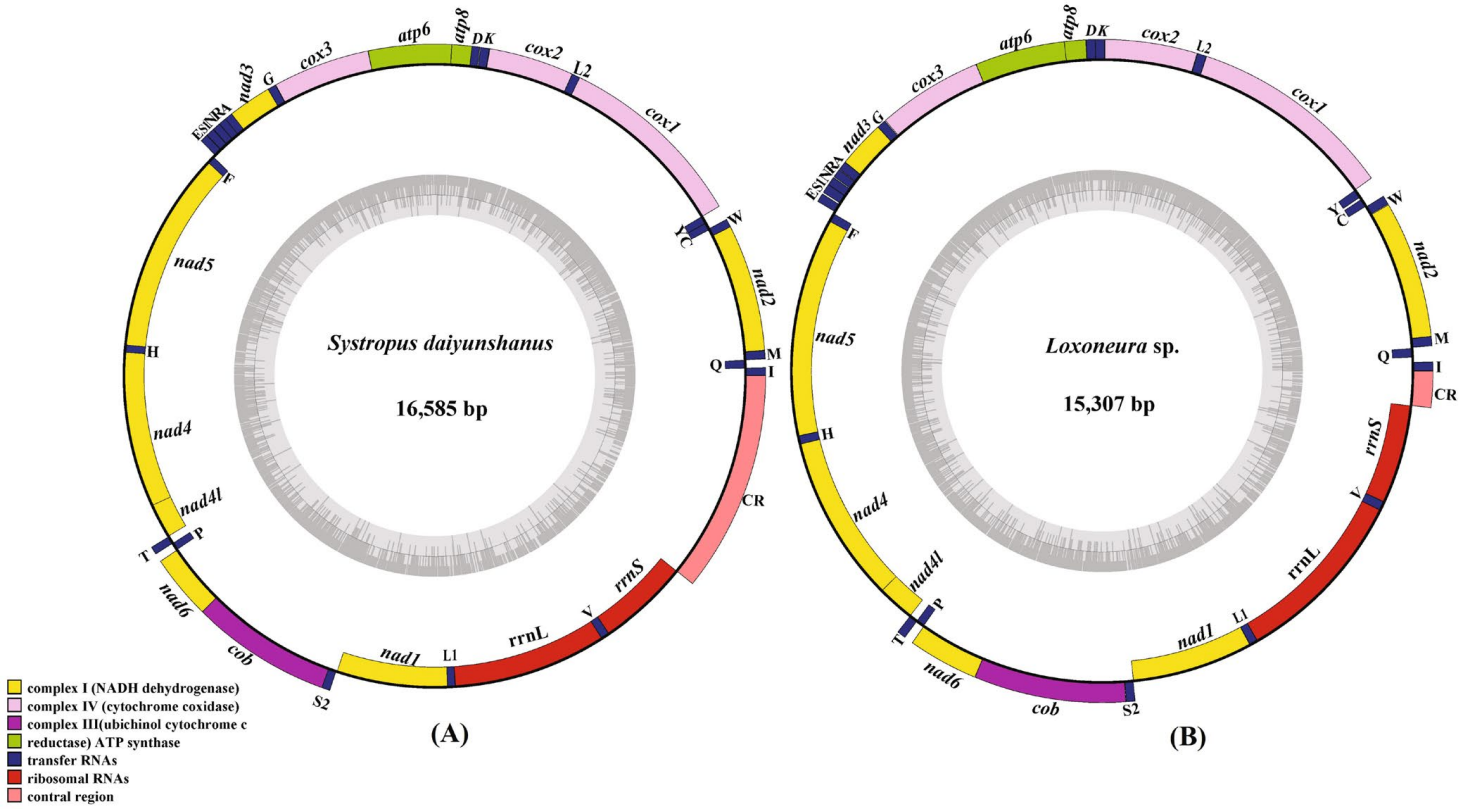
Mitogenome structure maps of *Systropus daiyunshanus* and *Loxoneura* sp. The genes on the outer circle are located on the majority strand, whereas the genes on the inner circle are located on the minority strand. The color‐filled deep blue indicates tRNAs, red indicates rRNA and CR, and the remaining colors indicate PCGs (yellow indicates nad genes, green indicates atp genes and purple indicates cob genes). The length of the graph on the circle represents the length of the gene. L1, L2, S1, and S2 represent the transfer RNAs (tRNAs), tRNA‐Leu (UAA), tRNA‐Leu (UAG), tRNA‐Ser (AGN), and tRNA‐Ser (UCN), respectively.

The set of 16 newly sequenced complete mitogenomes exhibits characteristics similar to those published dipteran mitogenomes. These mitogenomes possess a circular structure that is highly compact, showcasing a relatively conserved gene content. Each mitogenome is comprised of 37 genes, including 13 PCGs, 22 tRNA genes, two rRNA genes, and an A + T‐rich region known as CR, which is believed to function as the origin of the DNA replication region. Among the 13 PCGs, nine are located on the majority strand (J‐strand), while the remaining four PCGs, along with eight tRNAs and the two rRNAs, reside on the minority strand (N‐strand). The gene order and orientation in these mitogenomes remain consistent with the putative ancestral insect arrangement, as observed in the two representatives shown here (Figure 2). Notably, no gene rearrangement was observed through our mapping, and all tRNA genes and secondary structures were identified.

**FIGURE 3:**
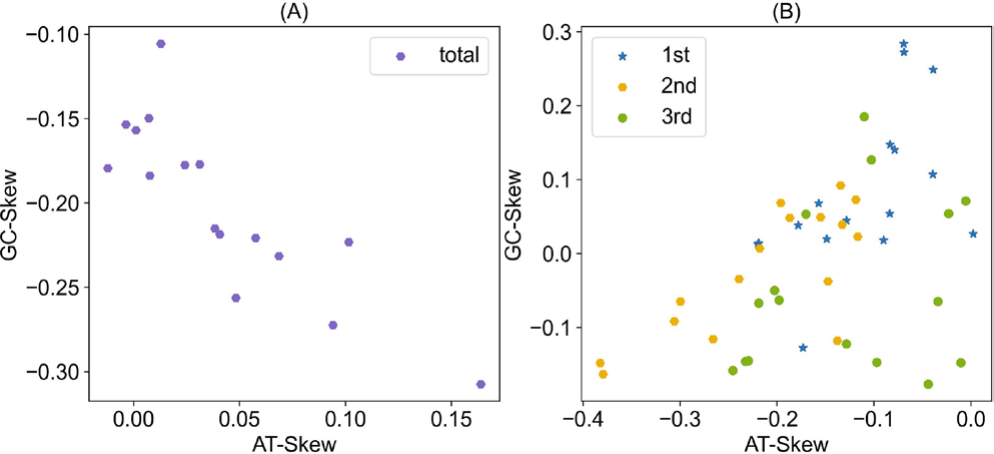
The scatter plot of AT‐skew and GC‐skew of 16 newly sequenced complete mitogenomes. (A) for all three codon positions of 13 PCGs, and (B) for 1st, 2nd and 3rd position, respectively.

**FIGURE 4:**
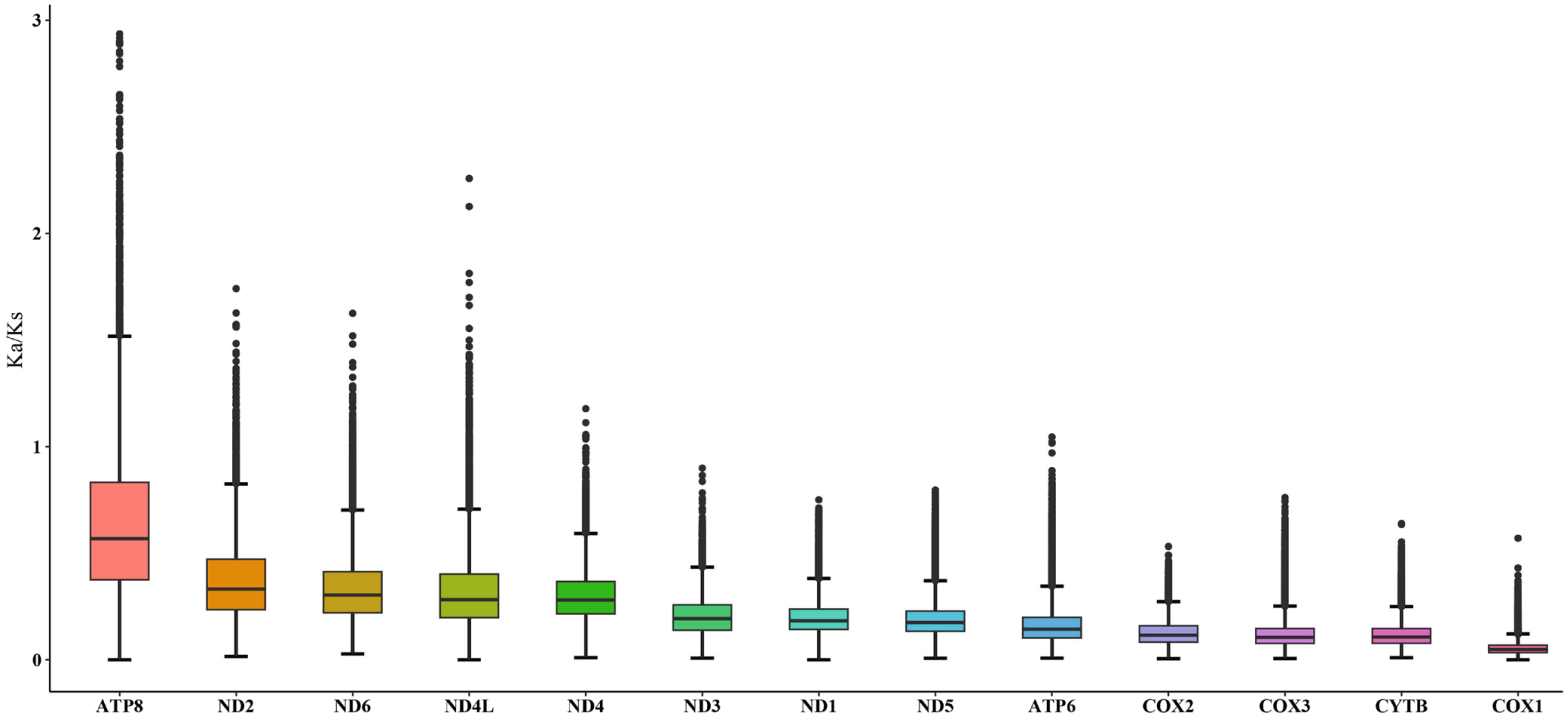
The evolutionary rates of 13 PCGs in the 131 Muscumorpha mitogenomes. Ka/Ks: The ratio of non‐synonymous nucleotide substitutions to synonymous nucleotide substitution. Neutral evolution (Ka/Ks = 1), purify selection (Ka/Ks < 1), positive selection (Ka/Ks > 1).

The 16 newly sequenced mitogenomes exhibited a range of full‐length sizes, spanning from 15,307 bp (*Loxoneura* sp.) to 16,585 bp (*Systropus daiyunshanus*) (Figure 2, Table 1), which falls within the typical range observed in other sequenced dipterans (14–20 kb). The variations in total length primarily result from differences in the size of the control region. A comparative analysis of A + T content across the sequenced mitogenomes revealed a range between 69.9% (*Clephydroneura* sp.) and 81.1% (*Asarkina porcina*). All nucleotide compositions exhibited a very strong bias (A% + T% > G% + C%) (Table 1). However, there were a few exceptions to this pattern. Three insects: *Lauxaniidae* sp. (Lauxaniidae), *Spaniocelyphus* sp. (Celyphidae), and *Asarkina porcina* (Syrphidae) showed slightly negative AT‐skew values (−0.1343, −0.0118, and −0.0037, respectively). In general, the newly sequenced mitogenomes displayed positive AT‐skew and negative GC‐skew, indicating a higher abundance of adenine (A) and cytosine (C) nucleotides (Figure 3A,B). For each PCG, the Ka/Ks ratio is less than one, and *ATP8* has the highest Ka/Ks ratio (0.66), followed by seven genes (*ND2*, *ND6*, *ND4L*, *ND4*, *ND3*, *ND1*, *ND5*) with Ka/Ks ratios of 0.37–0.20. *ATP6*, Complex III (*CYTB*) and Complex IV (*COX1*, *COX2* and *COX3*) have low Ka/Ks ratios with a range from 0.17 to 0.06 (Figure 4). These results imply all of these 13 PCGs experienced purifying selection, especially Complex III and Complex IV.

**TABLE 1:** Information summary of mitogenome of 16 newly sequenced species in Muscomorpha.

Family	Species	Length (bp)	A content (A%)	T content (T%)	G content (G%)	GC content (GC%)	AT‐Skew	GC‐Skew	Accession no.
Asilidae	*Clephydroneura* sp.	15,714	40.70	29.30	10.40	30.00	0.1629	−0.3067	MT424762
Lauxaniidae	*Homoneura* sp.1	16,208	38.60	38.00	9.60	23.50	0.0078	−0.1830	MT511108
	*Homoneura* sp.	16,284	38.90	38.30	9.70	22.80	0.0078	−0.1490	MT511111
	*Lauxaniidae* sp.	16,279	31.90	41.80	12.60	26.30	−0.1343	−0.0418	MT511112
Celyphidae	*Spaniocelyphus* sp.	15,342	37.60	38.50	9.80	23.90	−0.0118	−0.1799	MT511119
Syrphidae	*Phytomia zonata*	15,537	40.80	38.40	8.60	20.80	0.0303	−0.1731	MT511105
	*Asarkina porcina*	15,477	40.40	40.70	80.00	18.90	−0.0037	−0.1534	MT511106
	*Melanostoma* sp.	15,610	41.00	40.00	8.50	19.00	0.0123	−0.1053	MT511120
	*Microdon* sp.	15,770	42.00	38.10	7.40	19.90	0.0487	−0.2563	MT511101
Calliphoridae	*Chrysomya megacephala*	15,908	39.50	37.60	9.40	22.90	0.0246	−0.1790	MT511113
Sarcophagidae	*Blepharipa* sp.	15,835	41.40	38.40	7.90	20.20	0.0376	0.2178	MT511109
Tachinidae	*Tachinidae* sp.	16,291	40.90	37.70	8.40	21.50	0.0407	−0.2186	MT511123
Tephritidae	*Zeugodacus depressa*	16,546	39.70	32.60	10.20	28.10	0.0982	0.2740	MT477832
Platystomatidae	*Loxoneura* sp.	15,307	39.90	32.50	10.70	27.60	0.1022	0.2246	MT511102
Empididae	*Hercostomus potanini*	15,633	38.70	34.50	10.40	26.70	0.0574	0.2210	MT511125
Bombyliidae	*Systropus daiyunshanus*	16,585	39.60	34.50	9.90	25.80	0.0688	0.2326	MT511117

### 3.2 Matrix Heterogeneity and Completeness

**FIGURE 5:**
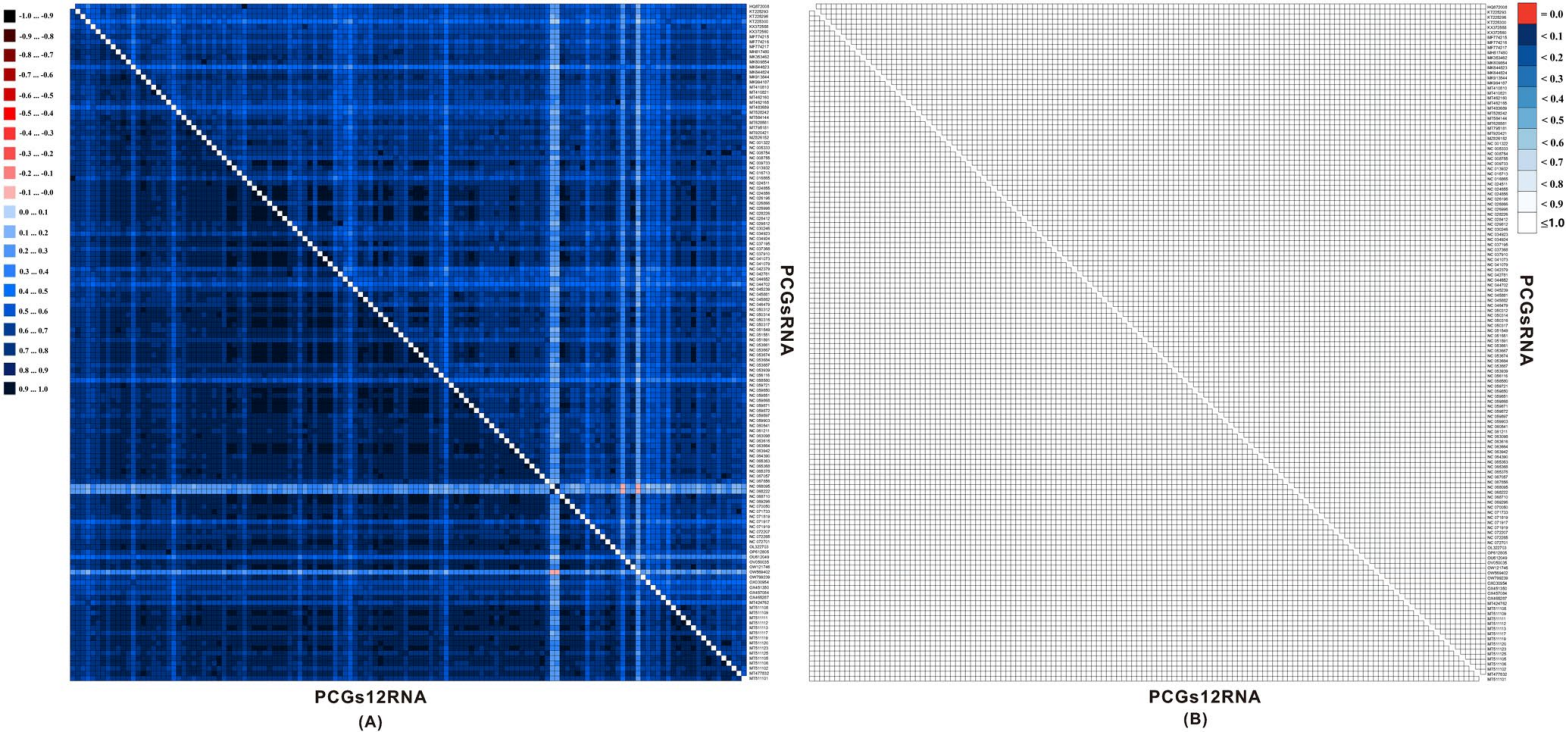
Heterogeneity between PCGsRNA and PCGs12RNA data sets in 131 species. (A) The mean similarity scores between sequences ranging from −1 in red to 1 in blue, calculated using AliGROOVE; (B) Completeness scores for pairs of sequences ranging from 0 to 1, calculated using AliStat. The upper right half plot indicates PCGsRNA, and the lower‐left half for PCGs12RNA. Species are denoted on the *Y*‐axis direction, and correspond to *X*‐axis in folding symmetry.

A heterogeneity test for the PCGsRNA and PCGs12RNA datasets utilized by the AliGROOVE procedure is a measure of sequence divergence heterogeneity, achieved through the performance of pairwise comparisons between all other sequences within a multiple sequence alignment. A heterogeneity test for the PCGsRNA and PCGs12RNA datasets revealed that two taxa from the Nyteribiidae (*Nycteribia parvula*: NC_068095, *Phthiridium szechuanum*: NC_068222) exhibited greater sequence divergence than that of one taxon from the Sciomyzidae (*Coremacera marginata*: OU612049) and one taxon from the Conopidae (*Thecophora atra*: OW569402). The divergence was flagged with red highlighting meaning lower similarity scores in the PCGsRNA dataset. While the red highlighting of Coremacera marginata has been eliminated in the PCGs12RNA dataset, the other taxa mentioned above have been not (Figure 5A). This indicates that the third codon positions in this analysis resulted in low heterogeneity, which was relatively alleviated in the PCGs12RNA dataset by removing the third codon positions. However, the effectiveness of this treatment was limited. Concurrently, the calculation of branch lengths in the ML tree also yielded a high score for the aforementioned species that may result in long‐branch attraction artifacts (LBA), which confirmed that heterogeneity is a factor of instability in phylogenetic relationship analysis (Table S6). Additionally, to quantify the completeness of the multiple sequence alignments, we also implemented the AliStat program to evaluate the completeness value of the two matrixes. The assessment of the completeness assessment of each matrix revealed that the nucleotide alignments had no non‐randomly distributed missing data (Figure 5B).

### 3.3 Phylogenetics of Muscomorpha

Here, we conducted a phylogenetic analysis based on ML under the partition model and the GHOST model for the nucleotide dataset and the PMSF model for the amino‐acid dataset, employing a dataset that included 131 representative species from 18 superfamilies of Muscomorpha with three species in Xylophagaidae as outgroup (Figures [Link], [Link], [Link], [Link], [Link], [Link]). Our sampling strategy ensured that all available families were covered (Figure S3). The Muscomorpha includes 18 superfamilies there: Nemestrinoidea, Asiloidea, Empidoidea, Platypezoidea, Conopoidea, Sciomyzoidea, Ephydroidea, Neriodiea, Lauxanioidea, Sphaeroceroidea, Tephritoidea, Diopsoidea, Carnoidea, Syrphoidea, Opomyzoidea, Hippoboscoidea, Oestroidea, Muscoidea. It is notable that, except for the superfamilies Neriodiea, Diopsoidea, Opomyzoidea, and Oestroidea have been demonstrated to be paraphyletic groups, the remaining superfamilies have been confirmed to be monophyletic.

The superfamily Muscoidea was robustly supported as monophyletic, and the four members' relationship within it was shown as ((Muscidae + Fanniidae) + (Scathophagidae + Anthomyiidae)), except Anthomyiidae, the other three families are monophyletic. The superfamily Oestroidea was found to be a paraphyletic group, Calliphoridae was proved to be monophyletic, while Sarcophagidae, Tachinidae, and Oestridae were all recognized as paraphyletic groups. This was due to Rhiniidae, Polleniidae, and Rhinophoridae each having a single representative, which resulted in some instability in their placement. The superfamily Hippoboscoidea was strongly supported as monophyletic, and the four members' relationship within it was manifested as (((Nycteribbidae + Streblidae) + Hippoboscidae) + Glossinidae). Four families, except Streblidae, were demonstrated to be monophyletic. However, the PCGs12RNA matrix with partition evolution model separates the Nycteribbidae and Streblidae families. The superfamily Ephydroidea was recovered as monophyletic with the member relationship (Drosophilidae + Ephydridae), and two families were also monophyletic. The superfamily Ephydroidea was formed as a sister group to the Calyptratae (the lineage comprised of Muscoidea, Oestroidea and Hippoboscoidea, which is a monophyly). The superfamily Tephritoidea was proved to be monophyletic, and the family members relationship was well supported as (((Platystomatidae + Tephritidae) + Ulidiidae) + (Piophilidae + Lonchaeidae)). The superfamily Lauxanioidea was found to be non‐monophyletic, as the Chamaemyiidae with a single sample here did not cluster a clade with the (Lauxaniidae + Celyphidae). In most of our analyses, the Lauxaniidae and Celyphidae were both monophyletic, but the Lauxaniidae did not recover monophyletic in the PCGs12RNA matrix. The Nerioidea was supported as monophyletic within Micropezidae sister to Cypselosomatidae. The family Psilidae of Diopsoidea did not cluster together with the Nothybidae, but was found to be near the Nerioidea, indicating that Diopsoidea was non‐monophyletic. The Sciomyzoidea is comprised of the Sciomyzidae, Dryomyzidae and Sepsidae in our analysis. All three families are monophyletic, with the latter two forming a clade separate from the former. Consequently, the Scimyzoidea is non‐monophyletic. The monophyly of Conopoidea depends on Conopidae, which was proved to be monophyletic in all topologies. The Opomyzoidea has not recovered the monophyly, but the four family members were monophyletic. This was evidenced by the fact that Clusiidae, Agromyzidae, Anthomyzidae and Fergusoninidae were each monophyletic. The latter two were a sister group. The Syrphoidea is composed of two monophyletic families, Syrphidae and Pipunculidae, but the Carnoidea was found to be nested within the Syrphoidea with moderate support, and thus became a sister group of the Syrphidae in most of our analysis. Surprisingly, in the AA matrix of the PCGs under the PMSF model, the Syrphoidea was recovered as monophyletic. The superfamily Platypezoidea was recovered as monophyletic just under the GHOST model of each matrix, although with poor support. Furthermore, the Platypezoidea was recognized as the sister group of the remaining Cyclorrhapha. The Empidoidea was non‐monophyletic, as the Dolichopodidae being united with a lineage with the Asiloidea leaving the Empididae as the sister group of the Hybotidae with strong support. Both families were monophyletic. The Asiloidea was portrayed as monophyletic solely in the PCGsRNA matrix under the partition model, due to the Therevidae having a single unstable sample in phylogenetic analyses. It is regrettable that the status of either the superfamily Asiloidea or Empidoidea as a basal group of the Muscomorpha is still unclear.

### 3.4 Divergence Time Estimation and Geographic Origin Tracing

**FIGURE 6:**
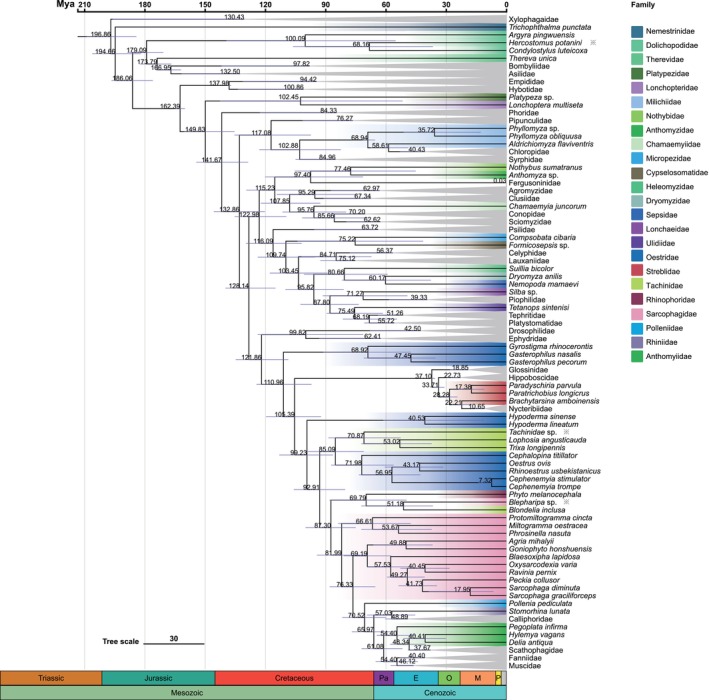
Divergence time of different clades in Muscomorpha inferred six fossil calibration points. The consensus tree presents the divergence time produced by the MCMC tree analysis of the PCGsRNA dataset using six fossil calibration points. Purple bars represent 95% credibility intervals for the ages of the clades. The numerical value above the purple bar represents the divergence of the corresponding node. A geological time scale is shown at the bottom.

The node dating resulting from the mcmctree based on the dataset PCGsRNA provides insights into the evolutionary history of Muscomorpha (Figure 6). It strongly supports that the origin of Muscomorpha occurred in the early Jurassic at 186.06 Mya [95% highest posterior density (HPD) 175.95–196.55 Mya]. The earliest diverging Muscomorphan lineages were the common ancestor of Empididae and Hybotidae at 162.39 Mya (95% HPD 159.99–164.99 Mya). Subsequently, the Platypezidae and Lonchopteridae diverged from the remaining muscomorphan lineages at 149.83 Mya (95% HPD 135.27–160.32 Mya). The vigorous diversity of muscomorphans took place during the Cretaceous period, particularly near the Cretaceous–Paleogene (K‐Pg) extinction event. A significant number of angiosperm fossils can be dated to the early Cretaceous, which means flowering plants provided an ample food source for insect pollinators during this period.

**FIGURE 7:**
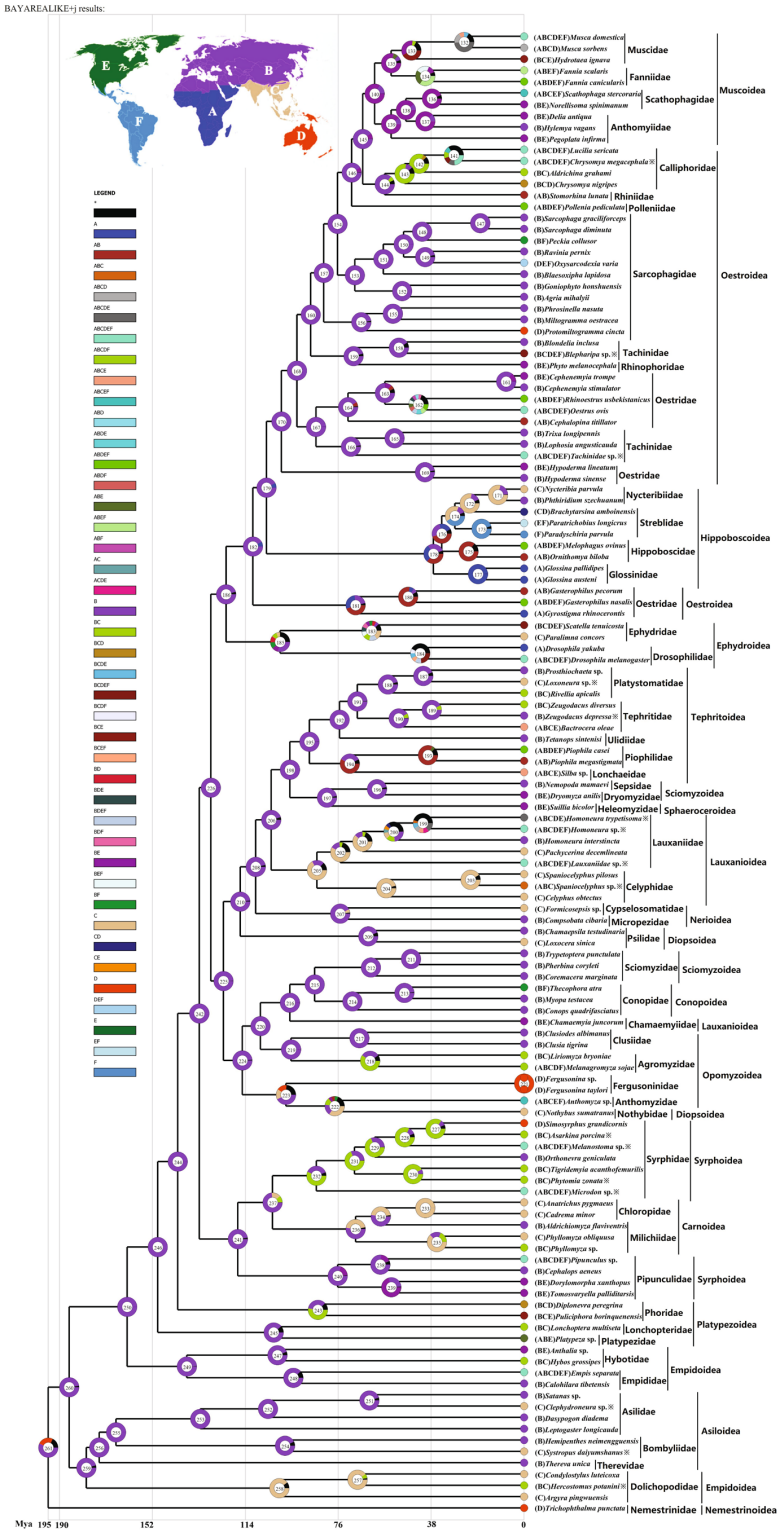
Geographical origin of muscomorph flies reconstructed from Bayarelike+J model implemented in RASP. Legend on the left indicates singular or combined biogeographic regions, with abbreviations A (Afrotropical region), B (Palaearctic region), C (Oriental region), D (Australasian), E (Nearctic region) and F (Neotropical region), respectively. Node colors reflect biogeographic designations (for species at tips) and ancestral‐area reconstructions (for internal nodes), probabilities are shown as color proportions for each node.

The ancestral‐area reconstruction based on our phylogenetic relationship points to either 56.9% probability Palaearctic region origin or 25.5% probability Australasian region origin of the ancestors of Muscomorpha (Figure 7, Data S1). The limited taxon sampling represents the superfamily Nemestrinoidea, which has been documented only in Australia. This superfamily forms the sister group to the remaining muscomorpha superfamilies. Most of the nodes on the tree were inferred as “speciation within areas,” which is defined as two descendants with the same range as the ancestor, that is, in situ diversification. Those superfamilies are of Palaearctic origin except for a partial clade of few superfamilies (Empidoidea, Carnoidea, Syphoidea, Lauxanioidea, Oestroidea) and a whole linage of few superfamilies (Platypezoidea, Hippoboscoidea, Muscoidea) with non‐Palaearctic origin. Dolichopodidae, as a member family of the Empidoidea, most likely originated in the Oriental region, which may be closely related to deep research in China. While the ancestor of the other clade of Empidoidea originated in Palaearctic region. It means that the early Empidoidea lineage might undergo a dispersal event from the Palaearctic to the Oriental region.

## 4 Discussion

### 4.1 Mitogenome Characteristics of Muscomorpha

A comparison of mitogenome sequences in the Muscomorpha shows that mitogenomes possess certain general features. Firstly, the gene composition and number of 16 newly sequenced mitogenomes encode 37 genes (13 PCGs, 22 tRNAs, and two rRNAs), with a similar gene arrangement and orientation to those previously reported in other Muscomorpha (Pu et al. 2017; Li, Yan, and Li 2023) (Figure 2). The mitogenomes are compact without introns, and the length variation mainly exists in the control regions. Secondly, the base composition and codon usage are identical to those of other published mitogenomes of Diptera (Guo et al. 2021; Ren et al. 2019, 2020). The AT and GC asymmetries known as A and T bases are more frequently used than G and C bases. And the codon usage shows a significant bias towards A and T (Figure 3). Thirdly, all tRNA genes can fold into the canonical clover‐leaf structure, except trnS2, which has lost the dihydrouridine (DHU) arm. Base pairs known as A‐Us and C‐Gs are occasionally used in our tRNAs. Furthermore, four kinds of mismatched base pairs are also found in the tRNA secondary structures: U‐G > U‐U > U‐A > U‐C (Figures S1 and S2).

### 4.2 Phylogenetic Relationships and Taxonomy

We inferred phylogenies within the Muscomorpha based on three kinds of evolutionary models for four data matrices (Table S7). The six trees produced similar results in the monophyly of the investigated superfamilies and families, but a difference was found in the relationships between the superfamilies and families. Muscomorpha, as a higher‐level lineage within Brachycera, was supported as a monophyletic group in six topologies. The monophyletic Nemestrinoidea and Asiloidea as basal clade of Muscomorpha are effectively addressed by the robust topology with PCGsRNA matrix through partition model ML analysis (Figure S3), which is consistent with previous research based on morphology alone (Yeates 2002) and combined data from 28S rDNA and morphology (Wiegmann et al. 2003), as well as supertree analysis (Yeates et al. 2007) and mitogenomic analysis (Song, Xi, and Yin 2022). The family Dolichopodidae as a member of Empidoidea is the sister group of Asiloidea, this attests that the monophyly of Empidoidea failed to recover, and that the remaining clade (Empididae + Hybotidae) of Empidoidea is the sister group of Cyclorrhapha in our analysis. The monophyletic Cyclorrhapha is well supported by our mitogenome data and agree in large part with many prior publications (Griffiths 1972; Wiegmann et al. 2011, 2003; Wood, Sinclair, and Cumming 1995; Song, Xi, and Yin 2022; Cameron et al. 2007). The sister group of Cyclorrhapha has been long disputed. In major of studies, the Empidoidea always appeared as a monophyletic group, sister to Cyclorrhapha, together termed Eremoneura. (Yeates and Wiegmann 1999; Wood, Sinclair, and Cumming 1995; Yeates 2002; Wiegmann et al. 2003; Sinclair and Cumming 2006; Yeates et al. 2007; Lambkin et al. 2012; Bayless et al. 2021; Wang et al. 2022). The alternative hypothesis is supported by multiple molecular plus morphological characters, indicating that the North American relict species *Apystomyia elinguis* Melander is the sister of Cyclorrhapha (Wiegmann et al. 2011; Shin et al. 2017). The discrepancy between the two hypotheses of the relationship may be due to a limited taxon sampling of Apystomyiidae, which is an amonotypic family with only one known extant species. We did not gain the mitogenome or sample of *Apystomyia elinguis* , leaving the question of whether Empidoidea or Apystomyiidae is the closest relatives of Cyclorrhapha still unsettled.

In the present study, the two lineages of Platypezoidea (Platypezidae + Lonchopteridae, Phoridae) are placed as the closest relatives of all other Cyclorrhapha. But the relationships among these three families members are inconsistent with the Phoroidea findings from the phylotranscriptomic analysis (Bayless et al. 2021). Additionally, such results clearly differ from those of studies that selected only one representative family for the superfamily (Platypezoidea or Phoroidea) was selected (Song, Xi, and Yin 2022; Wang et al. 2022). Subsequently, the Syrphoidea (Pipunculidae, Syrphidae) bifurcated within all other cyclorrhaphan groups, Schizophora. This relationship is well illustrated by diagnostic morphological traits of the schizophoran larval stage: in Schizophora a remnant of the larval head capsule is visible in dorsal view, otherwise, it is termed Aschiza (Griffiths 1972). Although the phylogenetic status of Syrphoidea was fixed, the monophyly here was not recovered due to the Carnoidea (Milichiidae, Chloropidae) being nested within. The considerable position of Carnoidea requires further sampling sequences to be provided, given that there are only two to three species from the two of 8–10 families in Carnoidea {Tree of Life Web Project (http://tolweb.org/tree/)}. Milichiidae here are paraphyletic as same as the recent mitogenomic analysis confirmed (Song, Xi, and Yin 2022).

The inconsistency in the intrafamilial relationships within Acalyptrtae across various datasets and methodologies still haunts us. In our partitioned maximum likelihood analysis, all Acalyptrtae families, with the exception of Ephydroidea, were classified into one to three monophyletic lineages distinct from Calyptrtae plus Ephydroidea (Figures S3, S5 and S7). This particular pattern, forming several primary clades, aligns to some extent with previous hypotheses (McAlpine 1989; Junqueira et al. 2004), even the comprehensive analysis sampled a substantial number of family members (Wiegmann et al. 2011). It is noteworthy that, there is insufficient family‐level coverage in previous investigations to discuss relationships within Acalyptrtae. In light of the explicit monophyly and taxonomic units of the Calyptrtae, numerous researches have focused on investigating the phylogenetic relationships within this group. Ephydroidea was supported as a sister group to Calyptratae only inferred by partitioned maximum likelihood analysis in our analysis. The Calyptrta phylogenetic hypothesis based on both morphological and molecular evidence supported as: ((Oestrioidea + Muscoid group) + Hippoboscoidea/Hippoboscid group) (Lambkin et al. 2012; Wiegmann et al. 2011; Bayless et al. 2021; Kutty et al. 2010, 2019). The relationship was stably maintained in our study. The monophyletic status of Hippoboscoidea has been a matter of contention in previous studies. In both the prior supertree analysis (Yeates and Wiegmann 1999) and the mitogenomic phylogeny analysis, the focus is on only two constituent families of Hippoboscoidea (Streblidae and Hippoboscidae) (Song, Xi, and Yin 2022) The monophyly of Hippoboscoidea has been demonstrated, while a combined quantitative characters and gene sequence data analysis proposed that Hippoboscoidea (Streblidae, Glossinidae and Hippoboscidae) was paraphyletic (Wiegmann et al. 2011). We recovered the monophyly of Hippoboscoidea and the presently recognized groups within it, including Glossinidae, Hippoboscidae, and Nycteribiidae, while Streblidae appears to be paraphyletic. Our relationships between the four constituent families (Glossinidae + (Hippoboscidae + (Streblidae + Nycteribiidae))) are well supported by a few molecular markers exploited by different phylogenetic methods before (Kutty et al. 2010; Petersen et al. 2007). The natural classification system of Oestroidea is complex, comprising between six to ten families (Yan, Pei, and Zhang 2021). The monophyletic status and phylogenetic relationships within the Oestroidea also have long been controversial (Song, Xi, and Yin 2022; Kutty et al. 2010, 2019; Yan, Pei, and Zhang 2021). These studies deciphering the phylogeny of Oestroidea emphasized different family members: Calliphoridae, Sarcophagidae, Tachinidae, Oestridae, Rhinophoridae, Rhinnidae, and Polleniidae are frequent at the core of the phylogenetic analysis. Of these, the first four families are particularly rich in species and command the greatest public attention. Calliphoridae was a polyphyletic termed calliphorid grade tested by four mitochondrial genes and four nuclear genes. This classification included three disparate subfamilies of Calliphoridae which were identified as the close relative of the Tachinidae and Rhinophoridae (Kutty et al. 2010). This monophyletic status is consistent with the latter molecular phylogeny, in which both mitochondrial and nuclear sequences were employed to infer that Calliphoridae is a polyphyletic group due to the phylogenetic position of Mesembrinellinae being uncertain. Unfortunately, the closest relative of Calliphoridae influenced by the phylogenetic methods is enigmatic (Song, Xi, and Yin 2022; Singh 2011; Junqueira et al. 2016). In consideration of the data integrity of each taxon and the balance of each taxonomic unit, the Calliphoridae with no Mesembrinellinae samplings in our analysis was recovered as monophyletic consistent with the phylogenomics analysis (Yan, Pape, et al. 2021). The sister group was identified as Rhiniidae proved by recent phylotranscriptomic analysis (Bayless et al. 2021). The monophyly of Sarcophagidae was well supported by a range of data sources, including mitochondrial and nuclear combined data (Kutty et al. 2010), transcriptome data (Yan, Buenaventura, et al. 2021), and mitochondrial data alone (Song, Xi, and Yin 2022; Junqueira et al. 2016). However, our work refuted former conclusions that Sarcophagidae was illustrated as paraphyletic. Previous analysis based on mitochondrial and nuclear data evaluated that Tachinidae were paraphyletic due to a single representative of Mesembrinellinae (Calliphoridae) and Oestridae separately nested (Kutty et al. 2010). While recent analysis based on transcriptomes and genomes confirmed that Tachinidae is monophyletic and is the sister group of Calliphoridae (Bayless et al. 2021; Kutty et al. 2019; Yan, Pape, et al. 2021). The phylogenetic analysis using four nuclear loci and large samplings also revealed Tachinidae and proposed Polleniinae (Calliphoridae *s.l*.) as the sister group (Stireman III et al. 2019). In our analysis Tachinidae and Oestridae both separate two clades, one of two clades with the two families united together, so Tachinidae and Oestridae were recognized as polyphyletic which is paralleled to prior findings (Song, Xi, and Yin 2022). However, the uncertainty remains regarding their sister groups, it is therefore clear that sampling selection whether including abundant species from the Mesembrinellinae (Calliphoridae), Polleniinae (Calliphoridae *s.l*.) and at the same time Oestridae is very much the key component in future attempts to attest the sister group of Tachinidae. Indeed, every other family of Oestrioidea has been hypothesized as its sister by at least one study (Wiegmann et al. 2011; Kutty et al. 2010; McAlpine 1989; Stireman III et al. 2019; Ding et al. 2015; Zhao et al. 2013). The relationship of Oestridae has varied considerably over time and has yet to reach a consensus. Morphological evidence including 118 characters supports that Oestridae including 25 Oestrid genera is monophyletic (Pape 2001). A comparative mitochondrial analysis at the subfamily level of Oestridae without other families hypothesized the same conclusion (Li et al. 2020). However, two phylogenetic analyses based on molecular data support the hypothesis that Oestridae is paraphyletic (Song, Xi, and Yin 2022; Stireman III et al. 2019). Oestridae in our analysis was recognized as polyphyletic. The limited sampling and sequencing data available for Rhinnidae and Polleniidae has resulted in their positions being temporarily closed to the Calliphoridae. The Muscoidea was ever confirmed as a paraphyletic group nested by Oestroidea family members based on mitochondrial and nuclear genes (Kutty et al. 2010, 2008) and transcriptome (Kutty et al. 2019). But in our analysis, we confirmed that Muscoidea ((Muscidae + Fanniidae) + (Scathophagidae + Anthomyiidae)) was monophyletic congruent with the conclusion based on a combination of morphological characters (McAlpine 1989).

Compared to previous phylogenetic analyses with limit taxonomic groups in Muscomorpha (Platypezoidea, Syrphoidea, Opomyzoidea, Sciomyzoidea, Tephritoidea, Ephydroidea, Hippoboscoidea, Oestroidea, and Muscoidea) (Woodley 1989; Wiegmann et al. 2011; Song, Xi, and Yin 2022), the present study not only includes all of the cyclorrhaphous Brachycera above noted but also increased the Empidoidea, Asiloidea, and Nemestrinoidea taxonomic range, which is congruent with previous studies. The most challenging of resolving phylogenies remains the dense taxonomic sampling and the degree of completeness of datasets, despite extensive taxon sampling employed in our analysis. Future research should concentrate on three primary areas: First, it is imperative to include additional families such as Apystomyiidae, Acroceridae, Apioceridae, Mydidae, Scenopinidae, Therevidae, Hilarmorphidae, Ironomyiidae, Sciadoceridae, and other Acalyptratae families. Second, it is crucial to incorporate these samples and extract molecular markers that can yield further evolutionary insights from the genome, thereby facilitating a more thorough investigation of phylogenetic relationships. Given the relatively low support observed at the base of the phylogenetic tree, subsequent efforts should aim to enhance the resolution of phylogenetic relationships at the basal position of Muscomorpha. Third, from the perspective of integrating diverse forms of evidence, future endeavors should aim to enhance the understanding of the evolution of adaptive traits, thereby addressing the potential limitations associated with an excessive dependence on molecular data.

### 4.3 Evolution of Muscomorpha

Our findings show that Muscomorpha may have diverged from the common ancestor of the Muscomorpha and Xylophagaidae at 196.86 Mya (the Early Jurassic), a period that coincides with the origin of many lineages of lower Branchycera (Wiegmann et al. 2011). This phylogenetic age was estimated earlier 24 Mya than the recent study by Song et al. (Song, Xi, and Yin 2022). In addition, the early divergence of Cyclorrhapha occurred at approximately 149.83 Mya (the Late Jurassic) coinciding with the estimates of Wiegmann et al. and Song et al. Abundant brachyceran fossils were explored in the Mesozoic, especially in the Late Jurassic and Early Cretaceous (Zhang and Wang 2017). The unit of Platypezidae and Lonchopteridae is the earliest diverging clade within Cyclorrhapha. The Platypezidae fossil records discovered in the late Jurassic perfectly matched the divergence time of Cyclorrhapha (Zhang, Yang, and Ren 2008).

Phylogenetic and molecular calibration showed that the common ancestor of Schizophora radiated into two major groups during the lower Cretaceous period (128.14 Mya). One group included major Acalyptrate families, while the other group included all Calyptrate families. Many extant families of Calyptrate originated near the K‐Pg boundary. A long gap between the Acalyptrate and Calyptrate has been proved in a previous paper. Furthermore, we did not find any reasonable fossil evidence from the lower Cretaceous and the early Paleogene (Wiegmann et al. 2011). The catalog of the fossil flies of the world listed many trace and amber fossils of Calyptrate from the Tertiary (Winkler et al. 2010; Evenhuis 2017; Michelsen 1996). Implying that a rapid lineage diversification in Calyptrate may take place before the Tertiary period near the K‐Pg extinction event. Coincidentally, the most recent common ancestor of extant Calyptratae lived was proved before the K‐Pg boundary (70 Mya) (Cerretti et al. 2017), which is 40 Mya later than our estimation (110.96 Mya). This period coincided with the diversification of the angiosperms to widespread the terrestrial world. And the angiosperm radiations provided new food resources and habitats and had a profound effect on flies (Zhang and Wang 2017). The Palaearctic region distribution of Muscomorpha is remarkable in our tracing. The geographical distribution scenario may have a close correlation with the present distribution of the samplings and the extent to which taxa have been studied in each area. Our taxon sampling has been widely studied in Asia. Therefore, more representative distributions need to be further investigated.

## 5 Conclusion

A comparison of mitogenome sequences in the Muscomorpha shows that mitogenomes reveal several general features. The gene composition and the number of 16 mitogenomes are identical to those of an ancestral dipteran mitogenome, and the lengths of these mitogenomes are similar to the ancestral ones also. Our phylogenetic analysis identified the Moscomorpha as a monophyletic group. The 14 of 18 muscomorphan superfamilies: Nemestrinoidea, Asiloidea, Empidoidea, Platypezoidea, Conopoidea, Sciomyzoidea, Ephydroidea, Lauxanioidea, Sphaeroceroidea, Tephritoidea, Carnoidea, Syrphoidea, Hippoboscoidea, Muscoidea, were confirmed to be monophyletic. But 4 of 18 superfamilies: Neriodiea, Diopsoidea, Opomyzoidea, and Oestroidea, were proved to be paraphyletic groups. Our analyses support an origin of the Muscomorpha in the Early Jurassic (196.86 Mya), which is considerably older than previous estimates nearly 20 years.

## Author Contributions

**Huan Yuan:** conceptualization (equal), software (equal), supervision (equal), validation (equal), writing – original draft (equal). **Wenbo Fu:** data curation (equal), software (equal), visualization (equal). **Shulin He:** investigation (equal), methodology (equal), supervision (equal). **Tingjing Li:** conceptualization (equal), methodology (equal). **Bin Chen:** conceptualization (equal), investigation (equal), methodology (equal), project administration (equal), resources (equal), software (equal), supervision (equal), validation (equal), writing – review and editing (equal).

## Conflicts of Interest

The authors declare no conflicts of interest.

## Supporting information


Data S1

Figure S1

Figure S2

Figure S3

Figure S4

Figure S5

Figure S6

Figure S7

Figure S8

Table S1

**Tables** **S2**‐**S7**

## Data Availability

All data are available as tables and figures in the main paper and its supporting information files. The raw data of newly sequenced samples of this study are openly available from the NCBI (https://www.ncbi.nlm.nih.gov/) under BioProject No. PRJNA1154623.
