# Supplementary figures and images for: Study of Mitogenomes Provides Implications for the Phylogenetics and Evolution of the Infraorder Muscomorpha in Diptera
Source: Ecol Evol. 2025 Jan 16;15(1):e70832. doi: 10.1002/ece3.70832 (PMC11739608; doi:10.1002/ece3.70832)

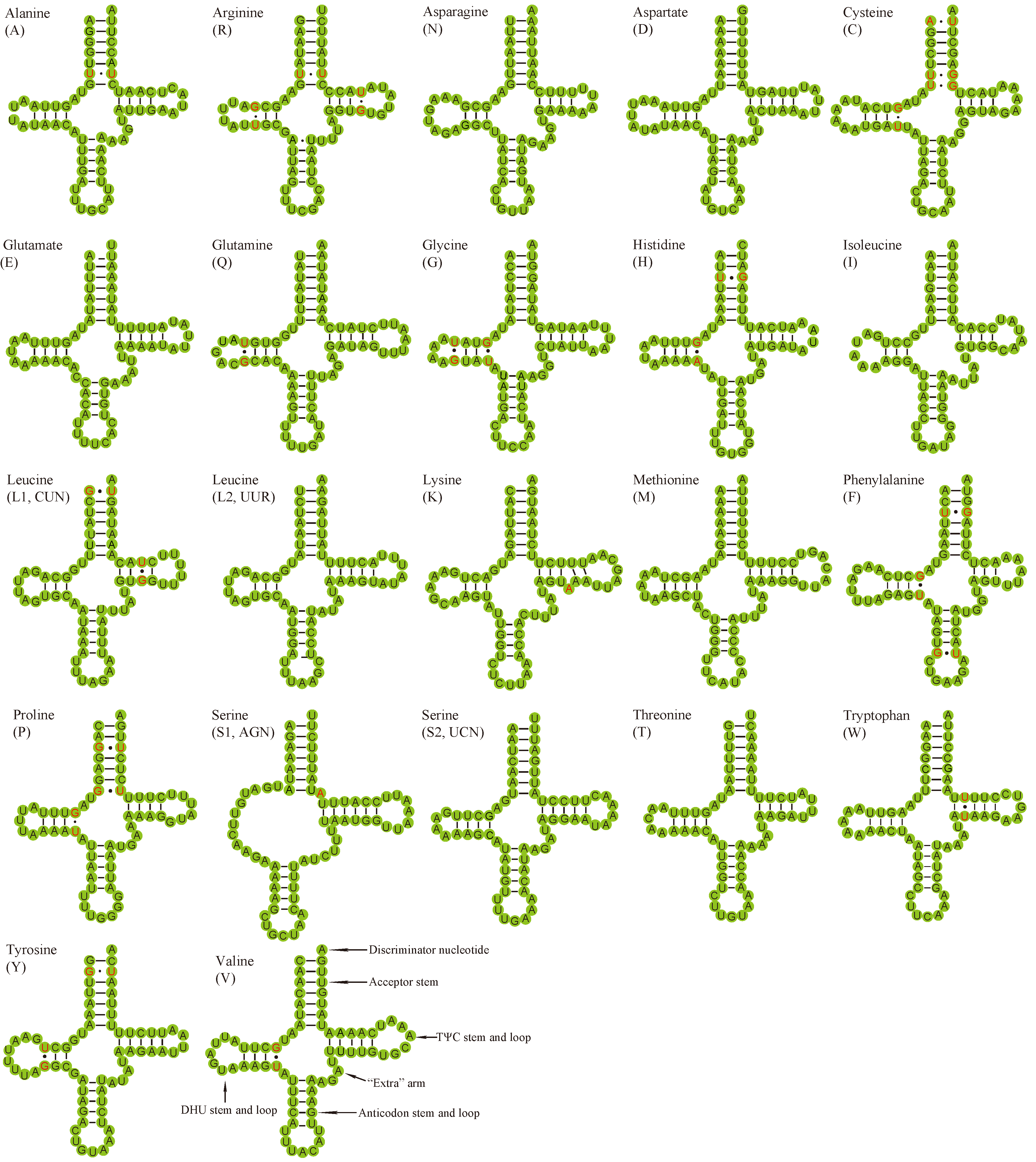

Supplement: Supplementary file 2 — Figure S1 [file ECE3-15-e70832-s011.tiff]

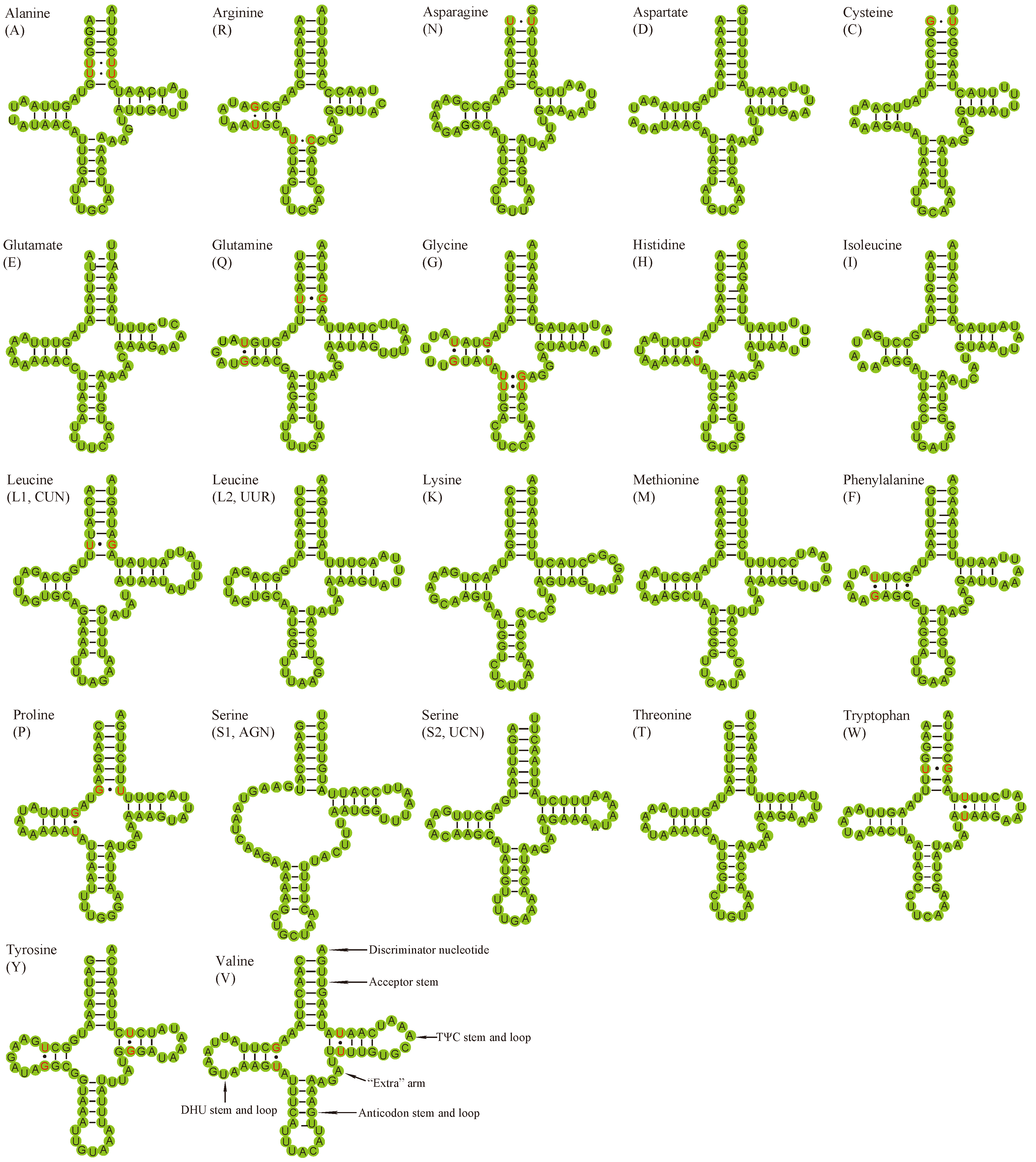

Supplement: Supplementary file 3 — Figure S2 [file ECE3-15-e70832-s002.tiff]

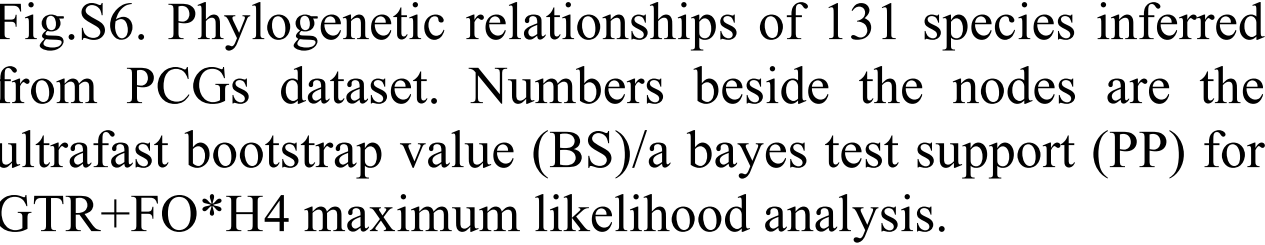

Supplement: Supplementary file 7 — Figure S6 [file ECE3-15-e70832-s008.pdf]
