# Supplementary material for: Study of Mitogenomes Provides Implications for the Phylogenetics and Evolution of the Infraorder Muscomorpha in Diptera
Source: Ecol Evol. 2025 Jan 16;15(1):e70832. doi: 10.1002/ece3.70832 (PMC11739608; doi:10.1002/ece3.70832)
Supplement: Supplementary file 8 — Figure S7 [file ECE3-15-e70832-s004.pdf]

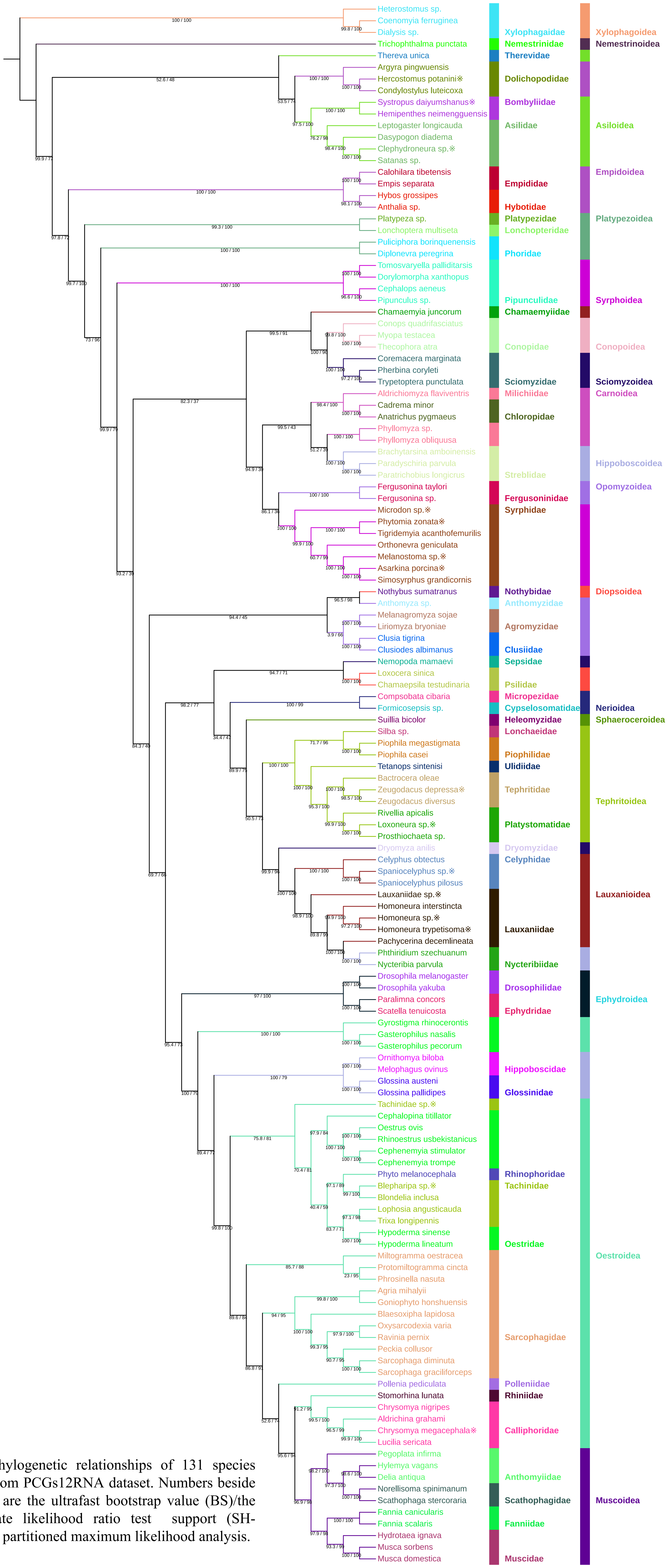

Fig.S7. Phylogenetic relationships of 131 species inferred from PCGs12RNA dataset. Numbers beside the nodes are the ultrafast bootstrap value (BS)/the approximate likelihood ratio test support (SH-aLRT) for partitioned maximum likelihood analysis.
