# Supplementary material for: Study of Mitogenomes Provides Implications for the Phylogenetics and Evolution of the Infraorder Muscomorpha in Diptera
Source: Ecol Evol. 2025 Jan 16;15(1):e70832. doi: 10.1002/ece3.70832 (PMC11739608; doi:10.1002/ece3.70832)
Supplement: Supplementary file 10 — Table S1 [file ECE3-15-e70832-s003.doc]

**Supplementary Table 1**. Collecte information of 16 newly sequenced species in Muscomorpha.

| Accession No | Organism | Place | Collection_date |
| --- | --- | --- | --- |
| MT424762 | *Clephydroneura* sp. | Minwen Village, Nasuo Town, Dongxing City, Guangxi | 10.Ⅷ.2017 |
| MT511108 | *Homoneura* sp1 | Wenxin Street, Caijia Town, Jiangjin District, Chongqing | 11.Ⅵ.2018 |
| MT511111 | *Homoneura* sp*.* | Shanwangping Village, Yuquan Town, Nanchuan District, Chongqing | 12.Ⅷ.2017 |
| MT511112 | *Lauxaniidae* sp*.* | Shentian Grassland, Beiping Township, Chengkou County, Chongqing City | 4.Ⅶ.2017 |
| MT511119 | *Spaniocelyphus* sp*.* | Bridge Village, Xiaonanhai Town, Qianjiang District, Chongqing | 13D.Ⅷ.2018 |
| MT511105 | *Phytomia zonata* | Changtou River, Huangying Town, Wulong District, Chongqing | 3D.Ⅷ.2018 |
| MT511106 | *Asarkina porcina* | Fuxing Village, Gaoguan Town, Chengkou County, Chongqing | 23.Ⅶ.2017 |
| MT511120 | *Melanostoma* sp. | Qingxigou, Caijia Town, Jiangjin, Chongqing | 13.Ⅵ.2018 |
| MT511101 | *Microdon* sp*.* | Wenxin Street, Caijia Town, Jiangjin District, Chongqing | 11.Ⅵ.2018 |
| MT511113 | *Chrysomya megacephala* | Dahonghai, Simian Mountain, Jiangjin District, Chongqing City | 8.Ⅵ.2018 |
| MT511109 | *Blepharipa* sp*.* | Dayi Neighborhood Committee, Dayou Town, Nanchuan District, Chongqing | 14.Ⅷ.2017 |
| MT511123 | *Tachinidae* sp*.* | Guaidun, Xincun Town, Wuyishan, Fujian | 16.Ⅹ.2018 |
| MT477832 | *Zeugodacus depressa* | Huanghe Village, Gaonan Town, Chengkou County, Chongqing City | 8.Ⅶ.2017 |
| MT511102 | *Loxoneura* sp*.* | Shiziping, Fuxing Street, Chengkou County, Chongqin | 30.Ⅵ.2017 |
| MT511125 | *Hercostomus potanini* | Naguo Village, Fulong Town, Fangchenggang City, Guangxi | 12.Ⅷ.2017 |
| MT511117 | Systropus daiyumshanus | Xiaonanhai, Qianjiang District, Chongqing | 12D.Ⅷ.2018 |
